# Nicotine dependence in Croatian male inpatients with schizophrenia

**DOI:** 10.1186/s12888-018-1606-1

**Published:** 2018-01-22

**Authors:** Marina Šagud, Bjanka Vuksan-Ćusa, Nenad Jakšić, Alma Mihaljević-Peleš, Maja Živković, Suzana Vlatković, Tea Prgić, Darko Marčinko, Wei Wang

**Affiliations:** 1 School of Medicine, University of Zagreb, Zagreb, Croatia; 2 Department of Psychiatry, University Hospital Center Zagreb, Kišpatićeva 12, 10 000 Zagreb, Croatia; 3 School of Medicine, University of Osijek, Osijek, Croatia; 4 School of Medicine, University of Mostar, Mostar, Bosnia and Herzegovina; 5 Psychiatric Clinic Vrapče, Zagreb, Croatia; 6 Department of Psychiatry, General Hospital Vinkovci, Vinkovci, Croatia; 7 Department of Clinical Psychology and Psychiatry, Zhejiang University College of Medicine, Hangzhou, China

**Keywords:** Nicotine dependence, Smoking behavior, Schizophrenia, FTND

## Abstract

**Background:**

Patients with schizophrenia have the highest known rates of cigarette smoking, but less is known about their smoking behavior and the differences across geographical regions, including Croatia.

The aim of this study was to compare patterns of nicotine dependence between patients with schizophrenia and healthy individuals, and to determine the relationship between clinical presentation and the severity of smoking.

**Methods:**

This cross-sectional study included 182 recently hospitalized male inpatients and 280 healthy males, who were daily smokers. All participants have fulfilled the Fagerstrom Test for Nicotine Dependence (FTND). Patients were also evaluated by the Positive and Negative Syndrome Scale (PANSS).

**Results:**

Patients had higher FTND total score (*p* = 0.010), smoked their first cigarette earlier in the morning (*p* = 0.000), consumed higher number of cigarettes (*p* = 0.000), while healthy subjects had more difficulties to refrain from smoking in places where it is forbidden (*p* = 0.000) and smoked more even when they were sick (*p* = 0.000). While severe dependence was more prevalent in the patient group, light dependence was more frequent in control subjects (*p* = 0.04). Smoking behavior was not associated with either PANSS total score or any of its subscales and items.

**Conclusions:**

Smokers with schizophrenia differ from healthy smokers in both smoking behavior and level of dependence. Longitudinal studies are needed to shed more light on the complex relationship between smoking and psychopathology in schizophrenia.

## Background

Smoking is one of the largest risk-factors for premature mortality from non-communicable diseases in general population [1]. Patients with schizophrenia have the highest known rates of cigarette smoking and it was consistently reported in different parts of the world [2–6]. Such high prevalence of smoking contributes to premature mortality in those patients, which does not lessen over time [7]. Men appear to be even at higher risk for those unfortunate findings compared to women [1, 7].

However, less is known whether the level of nicotine dependence differs in smokers with schizophrenia compared to healthy individuals, and how smoking interacts with symptoms of the disease. For example, among smokers who smoked at least 20 cigarettes per day, and had similar levels of nicotine dependence, those with schizophrenia or schizoaffective disorder had higher number of puffs smoked per cigarette, larger cigarette total puff volume, shorter inter-puff interval duration as well as higher carbon monoxide boosts, compared to participants who had no major mental illness [8]. Similarly, smokers with schizophrenia had higher four-minute nicotine boost and a higher dose of nicotine per cigarette than persons without mental illness in the previous year [9]. Moreover, male patients with schizophrenia had higher proportion of smoking more than 10 cigarettes daily, and lower proportion of individuals who stopped smoking [10] compared to males with negative history for psychiatric disorders. The relationship between illness severity and smoking indices is incompletely understood. Previous studies have reported mixed results on the association between smoking and the intensity of symptoms. While majority of studies suggested that smokers with schizophrenia had more severe symptoms [10–15], one study reported similar overall symptomatology [15], while a series of studies reported milder symptoms in smokers than in nonsmokers [16–18]. In patients with first-episode schizophrenia, smokers had higher positive and negative syndrome scale (PANSS) total score and PANSS positive subscale scores and similar PANSS negative and PANSS general scores, in comparison to patients who were nonsmokers [10]. Among patients with several psychiatric disorders, including schizophrenia, smokers were more likely than nonsmokers to report hallucinations, delusions and subjective thought disorder and had worse negative symptoms [11]. In patients with schizophrenia, smokers with severe nicotine dependence had greater PANSS positive, while smokers with mild nicotine dependence had higher PANSS negative scores, respectively, compared to patients who did not smoke [12]. In another study on patients with schizophrenia, smokers had higher PANSS positive score than nonsmokers, while tobacco smoking was associated with more positive symptomatology, and less negative symptoms. [13]. Moreover, PANSS total scores and the PANSS general psychopathology scores were higher in non-smokers compared to smokers while PANSS positive and negative scores were similar between smokers and non-smokers with schizophrenia [14]. In patients with treatment-resistant schizophrenia (TRS), smokers had higher PANSS total score and PANSS negative score than nonsmokers, while there were no differences in two groups in PANSS positive and general scores [15]. Smoking status was not associated with PANSS total score in a sample consisted of patients with schizophrenia and bipolar disorder, although no data on PANSS subscales were presented [5]. On the opposite, in male inpatients with schizophrenia, smokers had lower PANSS positive scores, with no differences in the PANSS total score or the negative and general psychopathology subscales compared with non-smokers [16]. Notably, in this study, smokers who smoked more cigarettes had lower intensity of negative symptoms [16]. In the two studies on male patients with schizophrenia, current smokers had lower PANSS negative scores than never smokers [17] and never smokers and former smokers [18]. The discrepancies across those studies might arise from many methodological issues, such as different measures of psychopathology, sample sizes, age and sex differences, study settings (outpatients versus inpatients) and the presence of comorbid substance abuse. For example, psychopathology was estimated by The Diagnostic Interview for Psychosis [11] and PANSS total score and its subscales [10] as well as only PANSS total score [5]. Of note, smokers with schizophrenia have also received higher daily doses of antipsychotics [12, 14] than nonsmokers. While larger doses are logically related to higher severity of symptoms, they could also result from smoking-induced hepatic metabolism of some antipsychotics, such as clozapine and olanzapine [19]. Interestingly, in the sample of patients with TRS, smokers and nonsmokers did not differ in terms of mean daily antipsychotic dose [15]. Majority of those studies analyzed the association of smoking versus non-smoking status with psychopathology [5, 10, 11, 14, 15, 13], while few have investigated the influence of severity of nicotine dependence on psychopathology [16].

The relationship between schizophrenia and nicotine dependence is complex [20] and includes some shared genetic vulnerability [21]. Reasons for such high prevalence of cigarette smoking in patients with schizophrenia are still unclear [20]. So far, dopamine appears to play a major role in this relationship. It is well-known that schizophrenia is linked to elevated dopamine levels in dorsal striatum and reduced cortical dopamine release [reviewed in [22]. Nicotine increases dopamine levels in striatum by stimulating its release via nicotinic receptors, and decreasing its degradation by inhibiting monoamine oxidase type A and B. [reviewed in [20]. Clinical consequences include behavioral stimulation, antidepressant effects and decrease of drug-induced extrapyramidal adverse events [reviewed in [20]. More recently, it has been reported that smoking also induced cortical dopamine release, at least in subjects who smoked ≥10 cigarettes daily and had no current psychiatric disorder [23].

There are differences across countries in both smoking prevalence and smoking behavior in general population [24], schizophrenic patients [25] and control samples [2]. For example, smoking prevalence in general population varied significantly across 18 European countries, ranging from 40.9% in Bulgaria to 16.3% in Sweden [26]. In Croatian general population the rate of current smoking was 26.6%, which was similar to European average of 27% [26] and to frequency of smoking in other European transitional countries [27].

Interestingly, smokers in countries with lower smoking prevalence such as Sweden, had higher nicotine dependence levels compared to those from countries with higher prevalence of smoking, such as Germany [24]. Healthy persons from United States had higher rates of heavy nicotine dependence compared to Spanish control sample [2].

Men had higher smoking prevalence than women, in European countries including Croatia [26], and unlike women, Croatian men did not show decreasing trend in smoking prevalence [27]. Croatian men also had higher prevalence of heavy smoking compared to women [28], which is in line with data from other regions. For example, in healthy persons, males had higher FTND total scores [24, 29], and higher nicotine and cotinine levels [29] than females. In patients with schizophrenia severe nicotine dependence was associated with male gender [30]. Patients with schizophrenia from Spain had higher smoking prevalence compared to those from Germany, Greece and Italy [25].

To the best of our knowledge, no study has compared smoking behavior in Croatian patients with schizophrenia and healthy individuals. Given the above gender and regional differences in smoking indices, and complex relationship between smoking and schizophrenia, more investigation is needed to verify factors associated with nicotine dependence in schizophrenia, in different countries. The aim of the present study was to contribute to the current knowledge of smoking behavior in schizophrenia, and to investigate smoking behavior in Croatian patients. Specific objectives were to:

1. compare patterns of nicotine dependence between patients with schizophrenia and healthy controls
2. determine the pattern between nicotine dependence and psychopathology in patients with schizophrenia

## Methods

### Study setting and subjects

This cross-sectional study was carried out in University Hospital Centre Zagreb, Department of Psychiatry, and Psychiatric Hospital Popovača. Inclusion criteria were: male inpatients aged 18 to 65 years, diagnosed with schizophrenia, who were current smokers. Since daily smoking is considered to be a sign of nicotine addiction [25], smokers were defined as individuals who declared to smoke at least one cigarette daily. Given the observed differences in smoking patterns observed in both healthy subjects [24, 29] and individuals with schizophrenia [25, 30] only males were enrolled. Exclusion criteria were:  intellectual disability and other conditions affecting the current capacity to participate in the evaluation, such as moderate to severe agitation and/or aggression, catatonic features, patients with first-episode psychosis, substance abuse and dependence in the previous year other than nicotine and caffeine dependence, and any severe and/or unstable comorbid somatic or neurological disorder. Although subjects with the most severe clinical presentations were not included because of insufficient cooperation and understanding of questions and purpose of the study, there were no cut-off values in overall disease severity for the exclusion of patients.

Incusion criteria for healthy group were: males who were daily smokers, and exclusion criteria were: current or previous psychiatric disorders, comorbid substance use (other than nicotine and caffeine), as well as somatic (inclusive neurological) disorders.

### Assessment

Diagnosis of schizophrenia was confirmed by the Structured Clinical Interview (SCID) [31] based on the DSM-IV criteria (American Psychiatric Association, 1994). The level of nicotine dependence was measured by the Fagerstrom Test for Nicotine Dependence (FTND) [32], which is composed of six questions about daily cigarette consumption. It assesses the extent of cigarette smoking and smoking behavior. This questionnaire is the most widely used measure of nicotine dependence [33] and is also a reliable tool for the assessment of smoking behavior in schizophrenia [34]. Both patients and control group have fulfilled the FTND. The Cronbach alpha coefficients in the healthy and patient group were 0.76 and 0.68, respectively, indicating adequate internal reliability (having in mind the low number of items comprising this scale).

Symptom severity in patients was evaluated by structured interview for PANSS, including the PANSS positive, PANSS negative, PANSS general psychopathology subscale [35] and PANSS Cognitive subscale [36]. The average total score was 114.35 (SD = 21.609).

For this study, participants were divided in three groups according to the levels of tobaccodependence: (1) smokers with low dependence (FTND 0–4) (2) smokers with moderatedependence (FTND = 5) and (3) smokers with severe dependence (FTND≥6).

The investigation was approved by local Ethics Committee and was carried out in accordance with the Helsinki declaration (1975). All patients have signed informed consent approved by Ethics Committee prior to inclusion. The investigation was a part of a project “Predictors of treatment response in schizophrenia”, BM1.45, sponsored by the University of Zagreb.

### Statistical analysis

We ran all the statistical analyses in the SPSS version 19 (SPSS, Chicago, IL). Descriptive statistics contained means (M) and standard deviations (SD). Internal reliability of the FTND was described by Cronbach alpha coefficients. Mann-Whitney U test, Chi-square test and independent samples t-test were used to examine the potential differences in age and tobacco dependence parameters between schizophrenia patients and healthy control subjects. Pearson correlations were computed to examine the zero-order relationship among the investigated variables. The level of statistical significance was set at *P <* 0.05.

## Results

Overall, 182 patients and 280 healthy individuals took part in this investigation. The average length of treatment was 17.49 years (SD = 8.395), while the average number of previous psychiatric hospitalizations was 5.91 (SD = 4.730).

The average age was 42.20 years (SD = 11.974) for patients and 40.26 years (SD = 10.250) for healthy participants. The difference in their average age was not statistically significant (*t* = 1.784, df = 436, *p* = 0.075).

The differences between the groups (patients and healthy controls) in the total score on the FTND scale, individual items as well as the three tobacco dependence categories, are shown in Tables 1 and 2.

**Table 1:** Comparisons between the schizophrenia patients and healthy controls in the investigated tobacco dependence parameters

	Schizophrenia patients (*n* = 182)	Healthy controls (*n* = 280)	U / X^2^(df)	*P*
FTND total (mean (SD))	5.60 (2.312)	4.98 (2.690)	21,911	**.010**
FTND item 1 (mean (SD))	2.07 (1.044)	1.59 (1.027)	17,951	**.000**
FTND item 2 (proportions of *yes* answers)	40.7%	60%	16.542 (1)	**.000**
FTND item 3 (proportions of *yes* answers)	70.3%	72.1%	0.178 (1)	.673
FTND item 4 (mean (SD))	1.55 (.857)	1.17 (.825)	19,227	**.000**
FTND item 5 (proportions of *yes* answers)	47.8%	38.9%	3.556 (1)	.060
FTND item 6 (proportions of *yes* answers)	40.1%	60.7%	18.782 (1)	**.000**

*FTND* Fagerstrom Test for Nicotine Dependence, *U* Mann Whitney test, *X*^*2*^*(df)* Chi-square test and degrees of freedom; Significant *P* values are highlighted in bold

**Table 2:** Frequencies of schizophrenia patients and healthy controls in the three tobacco dependence categories

	Mild tobacco dependence	Moderate tobacco dependence	Severe tobacco dependence
Patients with schizophrenia	58 (31.9%)	25 (13.7%)	99 (54.4%)
Healthy controls	119 (42.5%)	41 (14.6%)	120 (42.9%)

Chi-square test (X^2^) = 6.416; df = 2; *p* = 0.041

As presented in Table 1, patients had significantly higher total score on the tobacco dependence measure, compared to healthy subjects. On the individual items, patients scored higher on items 1 (they tended to smoke their first cigarette earlier after waking up) and 4 (they smoked more cigarettes a day). Higher percentage of healthy controls compared to patients found it harder to refrain from smoking in places where it is forbidden (item 2) and they smoked more even when they were sick (item 6). No differences were observed in items 3 and 5.

In addition, based on the chi-square test (*χ*^2^ = 6.416, *p* < .04) (Table 2) patients were more often categorized as severely dependent on tobacco, whereas healthy controls were more often in the low dependence category. There was no difference in the frequencies of moderate dependence category among groups. The exact distribution of patients and healthy controls in the three nicotine dependence categories is shown in Table 2.

Finally, there was no significant correlation between age and FTND total score in both groups, and there was also no correlation between the PANSS total score and positive and negative scores with FTND total score in the patient group (*p* > 0.05). However, negative correlations between item 2 on FTND scale and PANSS total score, as well as PANSS positive and general subscores, were observed, as presented in Table 3.

**Table 3:** Pearson correlations between FTND scores and values on the PANSS diagnostic scale

Variables	PANSS – total	PANSS – P	PANS S –N	PANSS – G
FTND 1	.04	.02	.04	.04
FTND 2	−.18*	−.17*	−.13	−.16*
FTND 3	−.03	−.09	.04	−.02
FTND 4	−.02	.05	−.04	−.06
FTND 5	−.08	−.09	−.09	−.03
FTND 6	−.02	.02	−.04	−.02
FTND total	−.06	−.05	−.04	−.05

*FTND* Fagerstrom Test for Nicotine Dependence, *PANSS* Positive and Negative Syndrome Scale *P* positive symptoms subscale, *N* negative symptoms subscale, *G* general psychopathology subscale

*statistically significant correlations (*P* < = .036)

## Discussion

Main findings of the present study are:

1. Smokers with schizophrenia had higher FTND score compared to healthy smokers,
2. Smokers with schizophrenia had higher rates of severe dependence while healthy smokers had higher rates of light dependence,
3. On the individual FTND items, patients with schizophrenia scored higher on items 1 and 4, healthy individuals scored higher on items 2 and 6, while there were no significant differences on items 3 and 5.

### Severity of nicotine dependence in patients and healthy controls

We confirmed previous reports of increased FTND scores in patients with schizophrenia compared to healthy individuals [34, 37] and increased frequency of heavy smoking compared with general population [3]. However, our findings disagree with similar prevalence of heavy smoking and FTND score in healthy persons and first-episode patients [10], and with even higher FTND scores in healthy controls [38]. The differences might be related to age, since the prevalence of smoking in patients with schizophrenia increases with age [5, 39]. Smokers with schizophrenia were older than nonsmokers [17]. Given the self-medication hypothesis of smoking in schizophrenia [20], it might be speculated that older patients smoked more often in order to alleviate extrapyramidal and depressive symptoms, which are related to longer disease duration [40, 41]. However, other studies on individuals with schizophrenia have reported that smokers were actually younger than nonsmokers [14, 16]. Because schizophrenia is highly heterogonous disorder, longitudinal studies are needed to investigate whether and how the pattern of smoking changes throughout its course.

The average age in both of our groups corresponds to age-range which had the highest prevalence of smoking in European general population [26]. The discrepancy with reference [38], might arise from differences in smoking limitation. While in the latter study there was a restriction of smoking, in our hospitals there is facility where patients are allowed to smoke. More smoking restrictions might discourage light smokers to consume cigarettes, but not individuals who are more heavily dependent [24].

In the present study, compared to healthy individuals, smokers with schizophrenia had also higher prevalence of severe, and lower prevalence of mild nicotine dependence. There is some evidence that severe nicotine dependence may have distinct biological changes [42, 43]. However, the present study did not measure biological markers.

### Differences in smoking behavior between patients and healthy individuals

Several differences in smoking behavior were observed between group of patients and healthy subjects.

### Item 1-How soon after you wake up do you smoke your first cigarette?

Our patients had higher scores on item 1, which has correlated with a difficulty in maintaining abstinence due to emerging withdrawal symptoms [44], and was repeatedly found related to nicotine dependence in general population [29], while data on patients with schizophrenia are missing. For example, FTND items 1, 4 and 5 were the best predictors of cotinine concentrations in general population [45]. This item was even proposed to be a good single-item measure of nicotine dependence [46]. Therefore, our findings smoking in European genre severe nicotine dependence in schizophrenia [3], particularly in males.

### Item 2-Do you find it difficult to refrain from smoking in places where it is forbidden?

While higher inhibition of smoking in patients compared to control group might appear counterintuitive, this finding could result from different settings. Our hospital facilities had smoking areas, and unlike healthy persons, patients had no smoking restrictions. On the contrary, smoking ban at workplace was found to decrease both average number of daily cigarette consumption and proportion of heavy smokers in Croatian healthcare stuff [47]. While majority of patients were unemployed and all healthy participants had regular jobs, those two groups might have been differently affected by smoke-free laws. Alternatively, higher levels of nicotine dependence were related to lower inhibitory capacity in healthy persons [48, 49] and individuals with different psychiatric conditions [49].

### Item 3-Which cigarette do you hate most to give up?

This item, which differentiates first morning cigarette from any other during the day, had similar scores in patients and controls. It is another FTND item focusing on morning smoking as an indicator of greater nicotine dependence, which is related to the preference of first cigarette in the morning. Some authors have raised concern that FTND may deflate nicotine dependence scores among smokers with schizophrenia [33]. Differences in restrictions to smoking between patients and controls also need to be taken into account in the interpretation of this finding.

### Item 4. The number of cigarettes, smoked per day

This item, together with item 1, is supposed to reflect the best nicotine dependence. The sum of those 2 items is referred to as the heaviness of smoking index [3, 50], and had the greatest impact on exposure to nicotine and carbon monoxide [51]. Patients in the present study had higher scores in both items 1 and 4, which is consistent with meta-analysis that patients with schizophrenia smoke “heavier” than smokers in general population [3] and with other reports of higher number of cigarettes smoked daily in patients in first-episode psychosis [52] and schizophrenia [53].

### Item 5. Do you smoke more frequently during the first hours after awakening than during the rest of the day?

No differences were found between groups on the item 5 score. Since both items 3 and 5 refer to higher priority of morning smoking compared to the rest of the day, both groups appear to have had similar levels of morning cigarette craving. Smokers with schizophrenia, however, use tobacco during the night, and therefore may maintain sufficient nicotine levels to suppress morning withdrawal symptoms [33]. Although the current study did not measure nighttime smoking, similar scores in items 3 and 5 might simply reflect less nocturnal smoking in healthy individuals.

### Item 6. Do you smoke even if you are so ill that you are in bed most of the day?

This item addresses persistence in maintaining nicotine concentrations during waking hours. In the present study, healthy persons scored higher on FTND items related to difficulties in restraining from smoking at certain places (item 2) and situations (item 6). It might be that, unlike healthy smokers, smokers with schizophrenia do not even attempt to refrain from smoking. In fact, patients with schizophrenia had impaired impulse control compared to healthy persons [54], and among patients, smokers exhibited even higher rates of impulsivity [55] and had different motives to smoke, such as “increased need of stimulation” and lower scores of “sociability” [56]. Therefore, variations in impulse control and motivation for smoking might help explain the higher score on items 2 and 6 in healthy controls.

### Nicotine dependence and severity of symptoms

We observed no associations between smoking severity and PANSS total score and any of its subscales or individual items. Those findings contrast previous reports of lower negative symptom severity in patients with higher levels of nicotine dependence [57–60] and positive correlations between FTND and PANSS negative scale, and several PANSS general items [61]. Our results are in line with other authors who observed no correlations between FTND total score [38], presence of severe nicotine dependence [30] and number of cigarettes per day [10], with any of PANSS items.

Those discrepancies might arise from disease severity, patient age, definitions of severity of nicotine dependence and comorbidity. Patients in majority of studies had the average PANSS total score lower than 60 [57–59], 80 [10, 30, 38], and 82 [60]. Our patients had more severe clinical presentation, as reflected by their PANSS total score of 114. The only study that also included acutely psychotic patients, with an average PANSS score of 105 [61], reported a positive correlation between FTND and PANSS negative scale, and items of unusual thought content, disorientation, poor attention, and poor impulse control [61]. While all those studies, including the current one, were cross-sectional, longitudinal studies are needed to determine whether the association between heaviness of smoking and severity of symptoms changes during phases of illness.

Another explanation might include gender, given that men had higher nicotine dependence in healthy individuals [24, 29] and in patients with schizophrenia [30], and other addictions. While cannabis and alcohol consumption were related to more positive symptoms [62, 63], and less prominent negative symptoms [62], those comorbidities might also influence severity of symptoms and contribute to discrepant findings. The definition of heavy smoking has also varied across studies. For example, [58] defined heavy smokers as those who smoked at least one pack of cigarettes per day, while we used FTND definition.

### Limitations

Due to cross-sectional design, causal relationship cannot be established. Groups were not controlled for other variables, such as education, financial status, and doses of antipsychotics. Smoking data were based on self-report and, given that patients were acutely psychotic, results might not be completely accurate. Given that PANSS rating was performed by 5 psychiatrists, who were in most cases also treating psychiatrists for those patients, potential bias cannot be excluded. In addition, given than only men without recent substance abuse were included, those findings might not be generalizable to women and / or patients with current substance abuse. Another limitation is that sociodemographic data for healthy control group were not collected.

## Conclusions

Our results extend previous findings of higher levels of nicotine dependence in patients with schizophrenia compared to healthy persons, to patients from Croatia, according to the hypothesis [25] that “schizophrenia is associated with increased smoking among smokers”. Our findings also highlight the differences in their smoking behavior compared to healthy smokers. In spite of those variations, degree of nicotine dependence was not associated with the severity of the disease or any particular symptoms. Given the high prevalence of both smoking and severe nicotine dependence in patients with schizophrenia, future investigations comparing smoking behavior in remitted patients versus those in acute relapse are needed.

## Abbreviations

FTND: Fagerstrom Test for Nicotine Dependence
PANSS: Positive and Negative Syndrome Scale
